# Sustained expression of MCP‐1 induced low wall shear stress loading in conjunction with turbulent flow on endothelial cells of intracranial aneurysm

**DOI:** 10.1111/jcmm.15868

**Published:** 2020-12-17

**Authors:** Cheng Chu, Gang Xu, Xiaocong Li, Zuowei Duan, Lihong Tao, Hongxia Cai, Ming Yang, Xinjiang Zhang, Bin Chen, Yanyu Zheng, Hongcan Shi, Xiaoyu Li

**Affiliations:** ^1^ Department of Neurology Affiliated Hospital of Yangzhou University Yangzhou University Yangzhou China; ^2^ Department of Pathophysiology Key Laboratory of Cardiovascular Disease and Molecular Intervention Nanjing Medical University Nanjing China

**Keywords:** IA, MCP‐1, miRNA, shear stress

## Abstract

Shear stress was reported to regulate the expression of AC007362, but its underlying mechanisms remain to be explored. In this study, to isolate endothelial cells of blood vessels, unruptured and ruptured intracranial aneurysm (IA) tissues were collected from IA patients. Subsequently, quantitative real‐time PCR (qRT‐PCR), Western blot and luciferase assay were performed to investigate the relationships between AC007362, miRNAs‐493 and monocyte chemoattractant protein‐1 (MCP‐1) in human umbilical vein endothelial cells (HUVECs) exposed to shear stress. Reduced representation bisulphite sequencing (RRBS) was performed to assess the level of DNA methylation in AC007362 promoter. Accordingly, AC007362 and *MCP‐1* were significantly up‐regulated while miR‐493 was significantly down‐regulated in HUVECs exposed to shear stress. AC007362 could suppress the miR‐493 expression and elevate the *MCP‐1* expression, and miR‐493 was shown to respectively target AC007362 and *MCP‐1*. Moreover, shear stress in HUVECs led to the down‐regulated DNA methyltransferase 1 (DNMT1), as well as the decreased DNA methylation level of AC007362 promoter. Similar results were also observed in ruptured IA tissues when compared with unruptured IA tissues. In conclusion, this study presented a deep insight into the operation of the regulatory network of AC007362, miR‐493 and *MCP‐1* upon shear stress. Under shear stress, the expression of AC007362 was enhanced by the inhibited promoter DNA methylation, while the expression of *MCP‐1* was enhanced by sponging the expression of miR‐493.

## 1 INTRODUCTION

Intracranial aneurysm (IA) is a type of cerebrovascular disorder featured by intracranial artery dilatation induced by endothelial layer defects.^1^, ^2^ The rupture of an IA leads to subarachnoid haemorrhage (SAH), a disease featured by high mortality as well as significant disability. The global incidence of IA is about 3%, although most patients with IA will not experience IA rupture during their life time.^3^, ^4^, ^5^

Correspondingly, it is of a significant value to accurately predict the chance of IA rupture. Haemodynamic characteristics play an essential role in the prognosis of IA. In particular, the shear stress applied to an IA is a key factor determining the physiological features of the arteries in the IA.^6^ In addition, the degeneration of IA wall starts from the apical surface of the IA, which is also the most common location of IA rupture. Moreover, the value of wall shear stress (WSS) is the minimum near the apex point of an IA.^7^ Furthermore, an IA with a narrow neck is more likely to generate a slow flow around the fundus region accompanied with a low and fluctuating WSS vector, which in turn can significantly change shear rate while accelerating the degeneration of IA wall.^8^, ^9^ A reduce blood flow velocity tends to cause thrombus while further expanding the dome of the IA.^10^, ^11^ Furthermore, a ruptured IA also tends to have a larger area of the aneurysm exposed to low WSS as compared to an unruptured IA.^12^

Long non‐coding RNAs (lncRNAs) are a class of non‐coding RNA with >200 nucleotides in size.^13^, ^14^, ^15^ In recent years, lncRNAs have been implicated in various cellular processes such as apoptosis, proliferation, invasion, and inflammation, which contributes to the pathogenesis of many disorders.^16^, ^17^, ^18^ Furthermore, emerging evidence suggests that lncRNAs play a critical role in the development of cardiovascular diseases.^19^, ^20^, ^21^

MCP‐1 is an inflammatory chemokine constitutively expressed by microglia, neurons, astrocytes, and microvascular endothelial cells. The expression of MCP‐1 is up‐regulated in traumatic brain damage, cerebral infarcts, as well as in many cognitive disorders.^22^, ^23^, ^24^, ^25^ It has been suggested that most of the MCP‐1 content in the body is synthesized by astrocytes. Upon binding to its receptor, MCP‐1 expressed in microglia can trigger their activation while promoting neuro‐inflammation, which in turn leads to cognitive disorders.^25^ In patients with IA, MCP‐1 can recruit and subsequently activate macrophages to attack blood vessel wall.^26^ Then, these macrophages will secret various pathological factors including proteinases as well as cytokines.^27^, ^28^ The role of MCP‐1 in IA pathogenesis has also been demonstrated in mice with MCP‐1 knock‐out.^27^, ^29^

It has been shown that AC007362 is deregulated upon shear stress and miR‐493 is a competing endogenous RNA of AC007362.^30^, ^31^ Furthermore, *MCP‐1* is a direct target gene of miR‐493. In addition, shear stress may suppress the methylation of promoters of many genes.^32^ Therefore, we hypothesized that shear stress may promote the expression of AC007362 by suppressing its methylation while enhancing the expression of *MCP‐1* by sponging the expression of miR‐493. To test this hypothesis, we set up a cellular model of shear stress and studied the effect of shear stress on the expression of genes involved in the signalling pathway of AC007362/miR‐493/*MCP‐1*. We also collected tissue samples from patients with ruptured small IA and unruptured big IA to compare their expression of AC007362/miR‐493/*MCP‐1*.

## 2 MATERIALS AND METHODS

### 2.1 Human subjects and sample collection

A total of 40 IA patients were recruited in our study. Then, the IA tissues from each patient were collected to isolate endothelial cells of blood vessels. Endothelial cells were isolated by utilizing a previously described protocol.^33^ In addition, depending on where the IA in each patient was ruptured or not, these patients were divided into two groups, that is a group of unruptured IA, in which the maximal diameter of IA is >4 cm, and a group of ruptured IA. In the next step, the demographic and clinicopathological characteristics of all participants, such as their age, sex, current status and past history of smoking, and comorbidities (such as hypertension, heart disease, high cholesterol, history of stroke, diabetes and osteoarthritis), were summarized and compared between the two groups using SPSS version 16.0 and Student's *t* test. The Human Research Ethics Committees of Affiliated Hospital of Yangzhou University has approved this research (Approval Number: YZDXFS153362) and all methods were performed in accordance with the last vision of the Declaration of Helsinki. Written informed consent was obtained from all patients before the initiation of this study.

### 2.2 Exposure of cultured endothelial cells to shear stress

The endothelial cells isolated from the blood vessels of IA tissues collected from the patients in both groups of unruptured IA and ruptured IA were seeded into tissue culture treated Petri dishes with a 0.1% gelatin surface coating. After the cells were 70%‐80% confluent, a flow apparatus (BD Bioscience) was employed to apply shear stress on the cells in accordance with the following protocol: On the day prior to the application of experimental shear stress, the cells were pre‐conditioned for 24 hours under 12 dyne/cm^2^ of a stable shear stress. Then, the value of shear stress was elevated to 35 dynes/cm^2^ generated by a non‐reversed flow to create a haemodynamic environment.

### 2.3 Reduced representation bisulphite sequencing (RRBS)

An RRBS analysis was carried out to determine the status of methylation of AC007362 promoter. In brief, HUVECs were divided into two groups, that is a control group and a shear stress group. Then, the cells in the shear stress group was subjected to the shear stress generated using the method described above, while the cells in the control group were not subjected to the stress. Similarly, the endothelial cells isolated from the blood vessels of IA tissues collected from the patients in groups of unruptured IA and ruptured IA were also exposed to the same shear stress, and the status of methylation of AC007362 promoter in these cells was analysed by utilizing a previously described protocol.^33^

### 2.4 RNA isolation and real‐time PCR

Total RNA in cultured HUVECs, as well as in the endothelial cells isolated from the blood vessels of IA tissues collected from the patients in both groups of unruptured IA and ruptured IA, was extracted following the conventional TRIzol method (Invitrogen). Then, the RNA isolated from each sample was converted to cDNA using reverse transcription carried out with an RT assay kit (Promega). In the next step, real‐time PCR was carried out using a TaqMan qPCR assay kit (Applied Biosystems) on a Light Cycler 480 real‐time PCR machine (Roche) following the procedures provided by the TaqMan kit manual to determine the relative expression of FTH1P10, RP11‐53P19, LINC00341, XLOC_040241, AF131215, XLOC_035542, RAMP2‐AS1, RP11‐690G19, RP11‐24F7, XLOC_023926, LINC00622 and RAB11B‐AS1, AC007362 and FTLP3. The calculation of relative expression was based on the routine 2^−ΔΔCt^ method and the expression of GAPDA was used as the internal reference to standardize the expression of target genes.

### 2.5 Cell culture and transfection

HUVECs were obtained from Type Culture Collection of the Chinese Academy of Sciences (Shanghai, China) and cultured in DMEM supplemented with 5% dextran (Sigma‐Aldrich), 2 mmol/L l‐glutamine, 8% heat inactivated foetal bovine serum, 100 μg/mL streptomycin and 100 U/mL penicillin (Gibco, Thermo Fisher Scientific). The tissue culture operation was carried out in a standard 37°C tissue culture incubator containing 5% CO_2_ and 95% air. When the cells reached about 80% confluence, they were randomly divided into 3 groups, that is an empty vector group, in which the cells were transfected with an empty pcDNA3.1 (+) vector, a AC007362 group, in which the cells were transfected with AC007362 mimics, and an anti‐miR‐493 group, in which the cells were transfected with miR‐493 siRNA. The transfection was carried out using Lipofectamine 3000 (Invitrogen) in accordance with the protocol of the manufacturer.

### 2.6 Vector construction, mutagenesis and luciferase assay

To determine the regulatory relationship between AC007362 and miR‐493 as well as between miR‐493 and *MCP‐1*, the sequences of AC007362 promoter and 3′ untranslated region (UTR) of *MCP‐1* containing miR‐493 binding sites were respectively cloned into pmiR‑REPORT luciferase vectors (Promega) to create wild‐type (WT) luciferase plasmids of AC007362 and *MCP‐1*. Then, site‐directed mutagenesis was performed to mutate the miR‐493 binding sites in the promoter of AC007362 and 3′ UTR of *MCP‐1*, respectively, and the mutated sequences were also cloned into pmiR‑REPORT luciferase vectors to create mutant (MUT) luciferase plasmids of AC007362 and *MCP‐1*, respectively. In the next step, HUVECs were plated into 24‐well plates and co‐transfected with WT or MUT plasmids of AC007362 or *MCP‐1* in conjunction with miR‐493 mimic or a scramble control using Lipofectamine 3000. At 24 hours after transfection, the luciferase activity of transfected HUVECs was assayed using a Dual Luciferase assay kit (Promega) following the protocol suggested by the manufacturer.

### 2.7 Western blot

Total protein in cultured HUVECs, as well as in the endothelial cells isolated from the blood vessels of IA tissues collected from the patients in both groups of unruptured IA and ruptured IA, was isolated using a RIPA lysis buffer (Thermo Fisher Scientific) and resolved by 12% SDS‐PAGE. Then, the resolved proteins were blotted onto a PVDF membrane (GE Healthcare), subsequently blocked using Tween‐20 containing 5% skim milk, treated at 4°C overnight with primary anti‐MCP‐1 and anti‐DNMT1 antibodies (1:5000, Santa Cruz Biotechnology), washed with Tris buffer, further incubated with HRP‐tagged secondary antibodies (1:1000, Cell Signalling), developed using an enhanced chemiluminescence kit (GE Healthcare) and finally analysed utilizing a GelDoc‐2000 imaging analysis system (Bio‐Rad) to determine the relative expression of MCP‐1 using GAPDH as the internal standard.

### 2.8 Statistical analysis

The levels of DNA methylation of the AC007362 promoter in different groups were compared using Student's *t* tests. A significance level of *P* ≤ .05 was used in this study. All continuous variables were compared using two‐sided Mann‐Whitney *U* tests, two‐sided Wilcoxon tests or two‐sided Student's *t* tests, while all qualitative data were compared using two‐sided Fisher's exact tests or two‐sided chi‐square tests. All statistical analyses were carried out with GraphPad Prism 5.0 software (GraphPad, Santa Barbara, CA).

## 3 RESULTS

### 3.1 Shear stress up‐regulated AC007362 and FTLP3 in HUVECs

**FIGURE 1:**
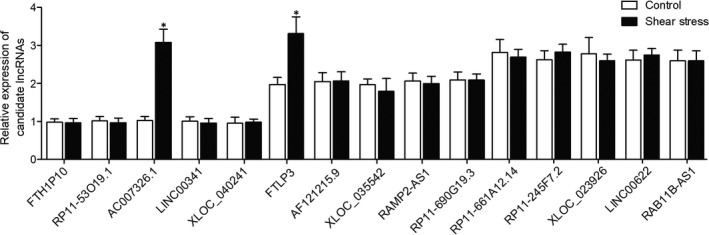
Expression of AC007362, FTLP3 was elevated in HUVECs exposed to shear stress, while no obvious changes were observed in the expression of FTH1P10, RP11‐53P19, LINC00341 and XLOC_040241, AF121215, XLOC_035542, RAMP2‐AS1 and RP11‐690G19, RP11‐690G19, RP11‐24F7, XLOC_023926, LINC00622 and RAB11B‐AS1 (*P value <.05 compared with control group)

Real‐time PCR was performed to check the expression of several lncRNAs in HUVECs exposed to shear stress. As shown in Figure 1, the expression of AC007362 and FTLP3 in HUVECs exposed to shear stress was significantly up‐regulated, although the increase in AC007362 expression was more pronounced than the increase in FTLP3 expression. Meanwhile, the expression of lncRNAs such as FTH1P10, RP11‐53P19, LINC00341, XLOC_040241, AF121215, XLOC_035542, RAMP2‐AS1, RP11‐690G19, RP11‐24F7, XLOC_023926, LINC00622 and RAB11B‐AS1 showed no obvious differences after exposure to shear stress.

### 3.2 Shear stress decreased miR‐493 and elevated MCP‐1 in HUVECs

**FIGURE 2:**
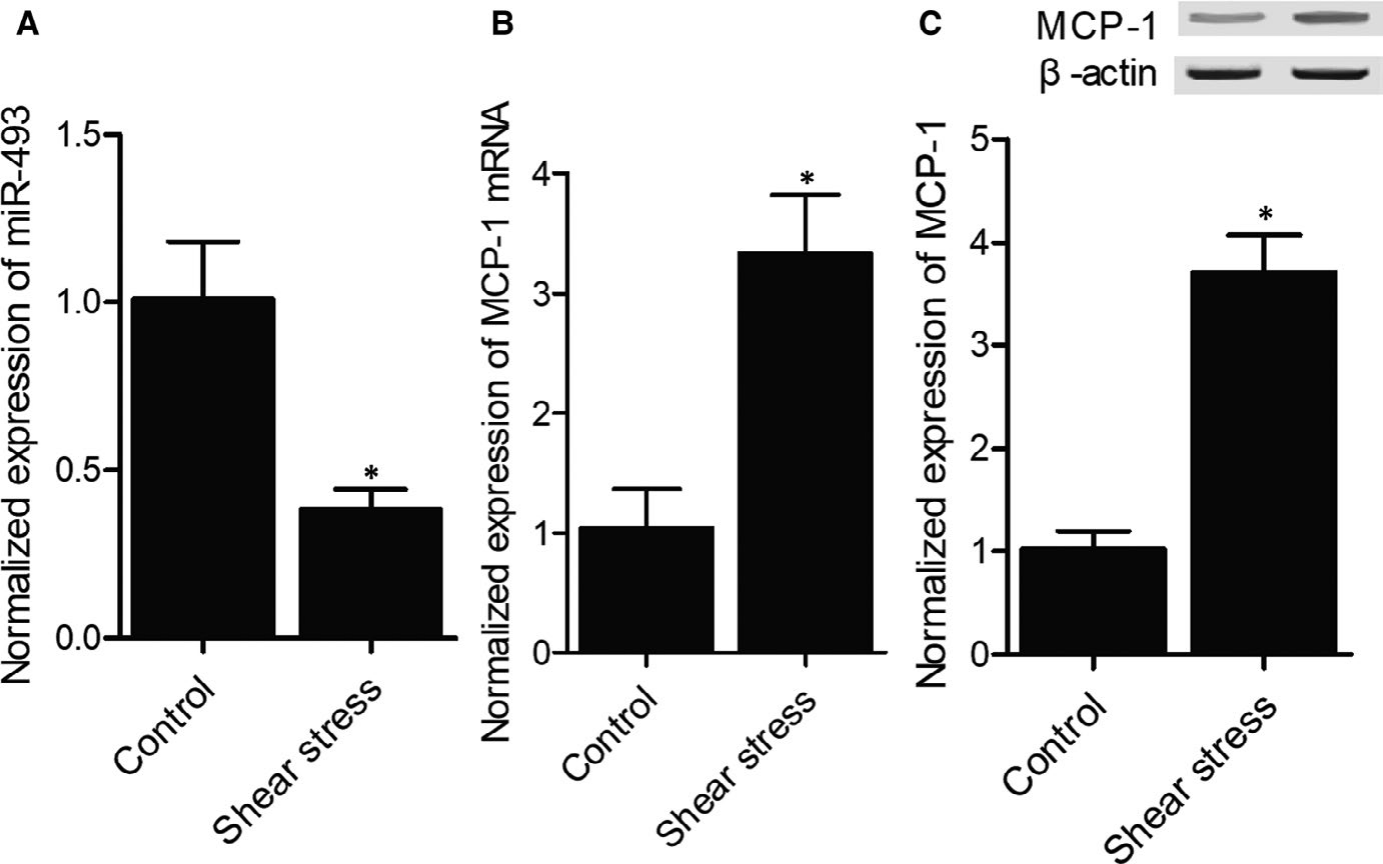
Shear stress altered the expression of miR‐493 and MCP‐1 (*P value <.05 compared with control group). (A) MiR‐493 expression was inhibited in HUVECs exposed to shear stress. (B) *MCP‐1* mRNA expression was enhanced in HUVECs exposed to shear stress. (C) Western blot showed that MCP‐1 expression was enhanced in HUVECs exposed to shear stress

MiR‐493 and MCP‐1 expression was analysed in HUVECs exposed to shear stress and the results showed that miR‐493 expression was dramatically down‐regulated by shear stress (Figure 2A). On the contrary, the expression of MCP‐1 in HUVECs was apparently elevated after the cells were exposed to shear stress (Figure 2B,C).

### 3.3 AC007362 decreased miR‐493 and elevated MCP‐1 in HUVECs

**FIGURE 3:**
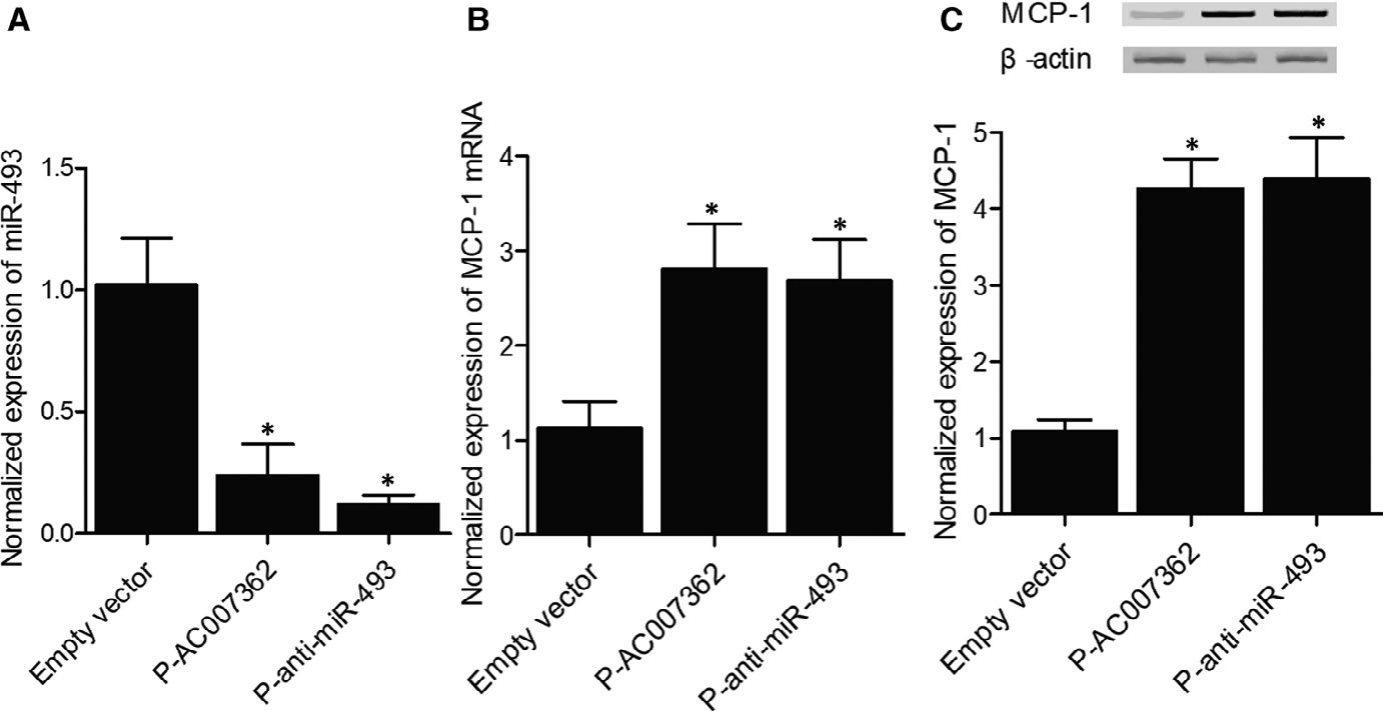
Overexpression of AC007362 affected the expression of miR‐493 and MCP‐1 (*P value <.05 compared with empty vector group). (A) MiR‐493 expression was inhibited in HUVECs transfected with AC007362 and anti‐miR‐493. (B) *MCP‐1* mRNA expression was enhanced in HUVECs transfected with AC007362 and anti‐miR‐493. (C) MCP‐1 protein expression was elevated in HUVECs transfected with AC007362 and anti‐miR‐493

AC007362 was transfected into HUVECs to evaluate its effect on the expression of miR‐493 and MCP‐1. MiR‐493 expression was notably decreased in HUVECs transfected with AC007362 and anti‐miR‐493 (Figure 3A), while the expression of MCP‐1 was evidently elevated after the transfection with AC007362 and anti‐miR‐493 (Figure 3B,C). These results implied that AC007362 could stimulate the expression of miR‐493 and MCP‐1.

### 3.4 MiR‐493 could bind to AC007362 and *MCP‐1*

**FIGURE 4:**
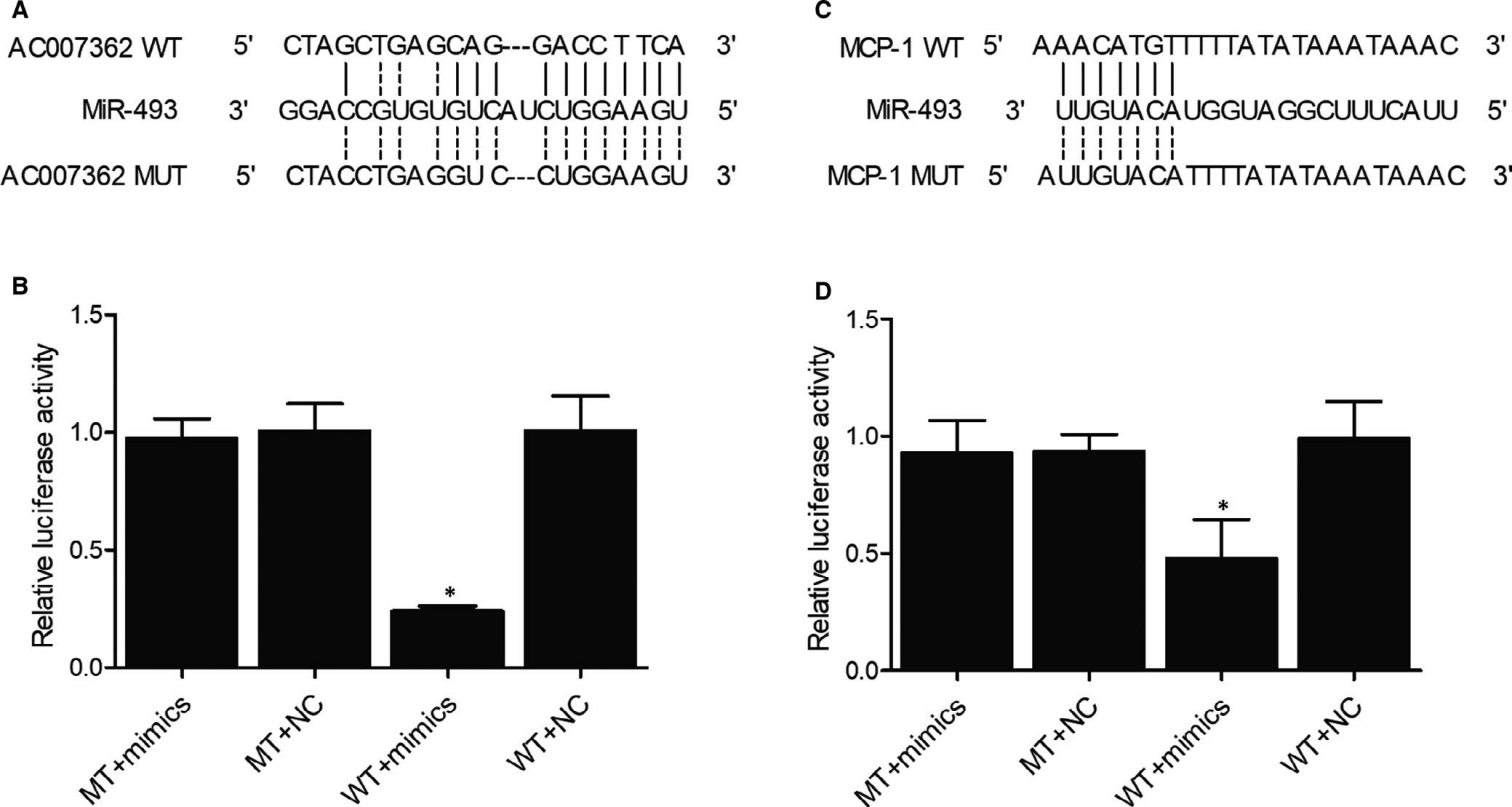
Binding of miR‐493 to AC007362 and *MCP‐1* (*P value <.05 compared with MT + mimics group). (A) Sequence analysis revealed a potential miR‐493 binding site on AC007362. (B) Luciferase activity of WT AC007362 was suppressed by miR‐493 mimics. (C) Sequence analysis revealed a potential miR‐493 binding site on *MCP‐1*. (D) Luciferase activity of WT *MCP‐1* was inhibited by miR‐493 mimics

An online bioinformatic analysis (TargetScan, http://www.targetscan.org/vert_71/) of potential targets of miR‐493 showed the presence of a miR‐493 binding site in AC007362 (Figure 4A). Then, luciferase vectors containing WT (Wild Type) or MT (Mutant) AC007362 were co‐transfected to the cells with miR‐493 mimics, and the luciferase activity of WT AC007362 was significantly inhibited by the transfection with miR‐493 mimics (Figure 4B). Similarly, the binding of miR‐493 to the 3′ UTR of *MCP‐1* (Figure 4C) was confirmed by the luciferase assay (Figure 4D).

### 3.5 Shear stress reduced DNA methylation of AC007362 promoter in HUVECs

**FIGURE 5:**
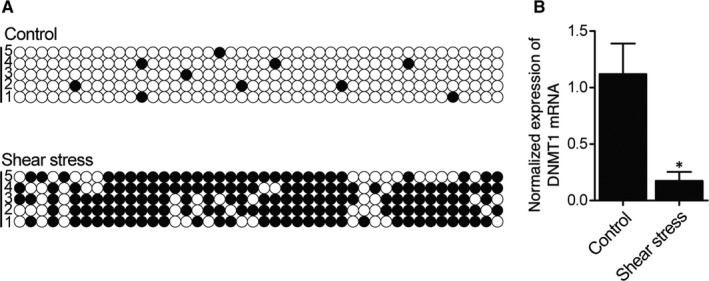
Shear stress reduced the DNA methylation of AC007362 promoter (*P value <.05 compared with control group). (A) DNA methylation of AC007362 promoter was decreased in HUVECs exposed to shear stress. (B) Shear stress down‐regulated DNMT1 mRNA expression in HUVECs

As shear stress was reported to affect DNA methylation, RRBS was performed here to study the effect of shear stress on the level of DNA methylation in the promoters of several genes including AC007362. In HUVECs, the exposure to shear stress remarkably suppressed the DNA methylation of AC007362 promoter (Figure 5A). In addition, as shown in Figure 5B, DNMT1 mRNA expression was dramatically decreased after exposure to shear stress and this result was presumably caused by the change in DNA methylation status.

### 3.6 DNA methylation was increased in the endothelial cells collected from unruptured IA

**FIGURE 6:**
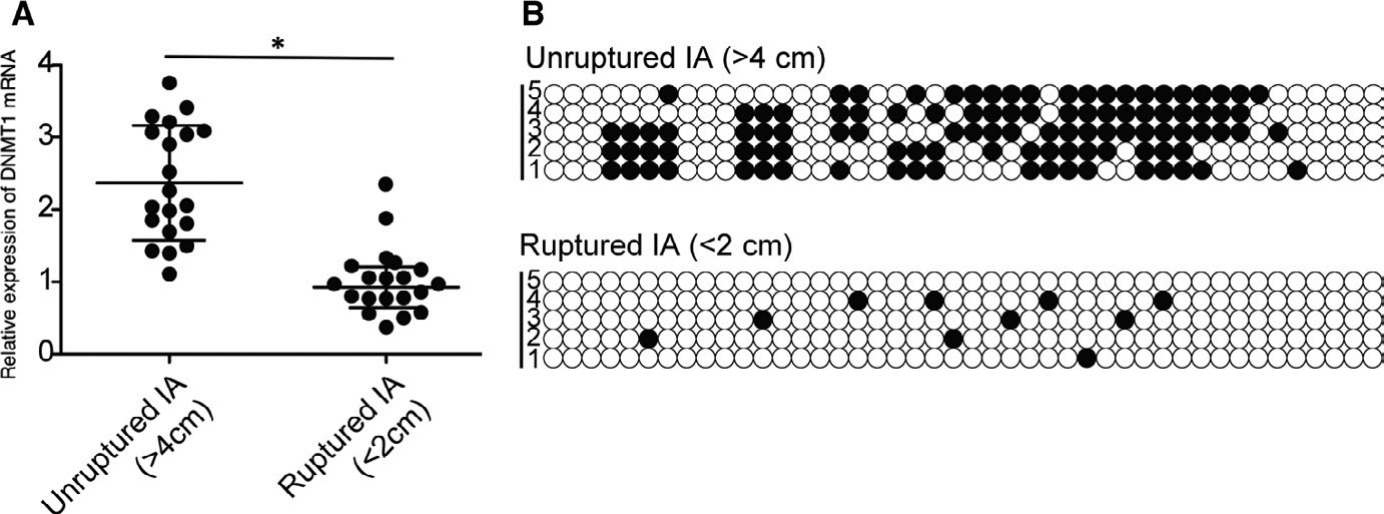
IA rupture decreased the level of DNA methylation of AC007362 promoter (*P value <.05 compared with unruptured IA group). (A) Decreased DNMT1 mRNA expression was observed in endothelial cells isolated from ruptured IA. (B) Reduced level of DNA methylation of AC007362 promoter was observed in endothelial cells isolated from ruptured IA

A group of IA patients were recruited into our study and then divided into unrultured IA group and ruptured IA group according to the presence of IA rupture. As summarized in Table 1, no difference was observed in respect to demographic, clinicopathological and genotypic parameters of between the different groups of IA patients. Then, endothelial cells were carefully isolated from the IA in the patients of the two groups to compare their levels of DNA methylation of the AC007362 promoter as well as the expression of DNMT1 mRNA. The results showed that DNMT1 mRNA expression and the DNA methylation of AC007362 promoter were both remarkably elevated in the group of unruptured IA (Figure 6A,B).

**TABLE 1:** Demographic and clinicopathological characteristics of the participants of this study

Characteristics	Unruptured IA (>4 cm) (n = 20)	Ruptured IA (<2 cm) (n = 20)	*P* value
Age	62.5 ± 5.6	63.1 ± 6.2	.536
Age (median (Q1/Q3))	60 (53.3/73.6)	63 (56.2/71.6)	.684
Sex (female, %)	12 (0.6)	13 (0.65)	.964
Smoker (%)	3 (0.15)	1 (0.05)	.854
Comorbidities (%)			.684
Hypertension	11 (0.55)	12 (0.6)	
Heart disease	3 (0.15)	2 (0.10)	
High cholesterol	5 (0.25)	5 (0.25)	
Stroke history	1 (0.05)	2 (0.10)	
Diabetes	8 (0.40)	10 (0.50)	
Osteoarthritis	2 (0.10)	1 (0.05)	

### 3.7 MCP‐1 and AC007362 were elevated and miR‐493 was inhibited in ruptured IA tissues

**FIGURE 7:**
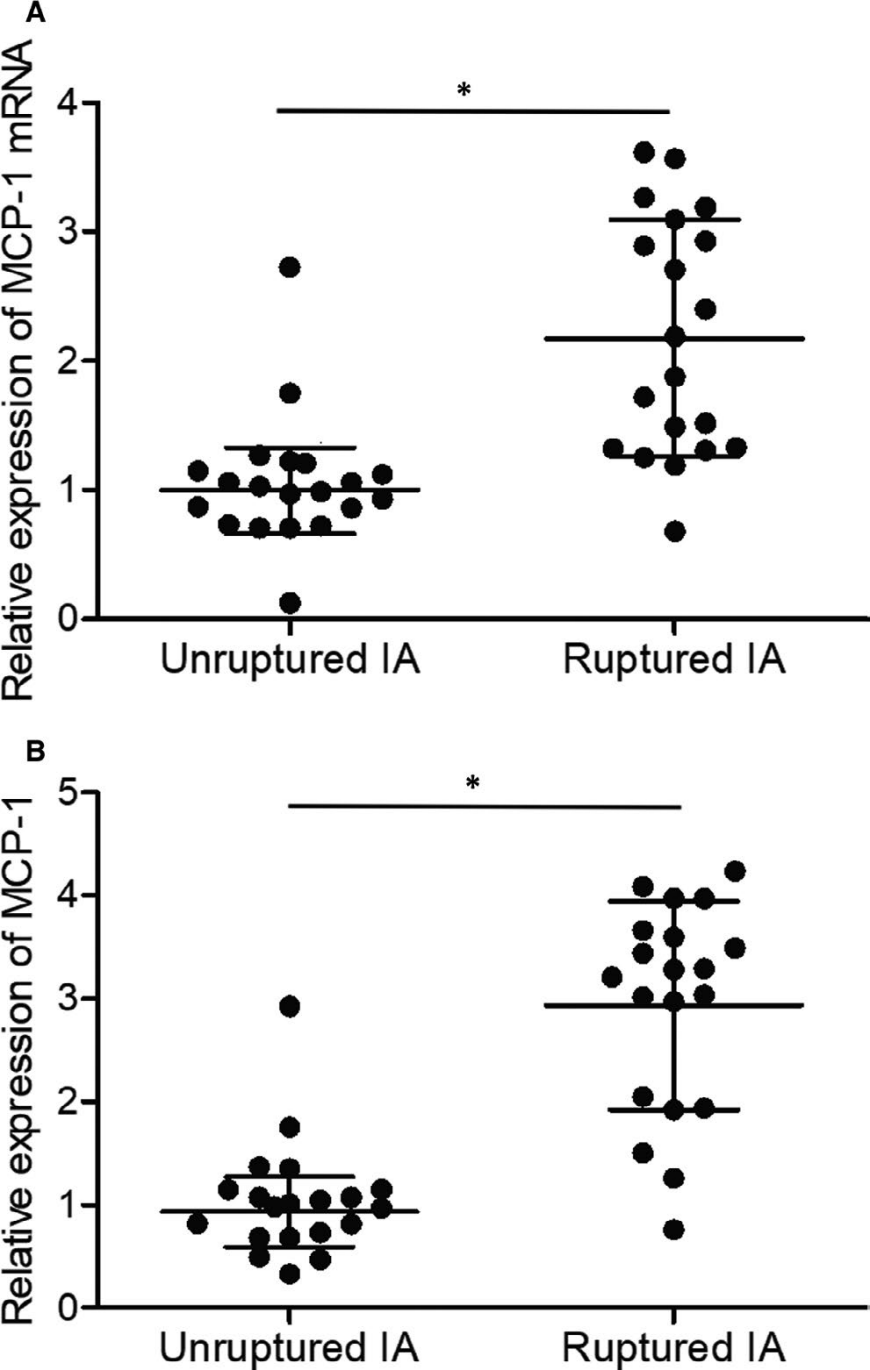
Differential expression of MCP‐1 in endothelial cells isolated from unruptured and ruptured IA (*P value <.05 compared with unruptured IA group). (A) *MCP‐1* mRNA expression was elevated in endothelial cells isolated from ruptured IA. (B) Plasma MCP‐1 protein expression was enhanced in ruptured IA group

**FIGURE 8:**
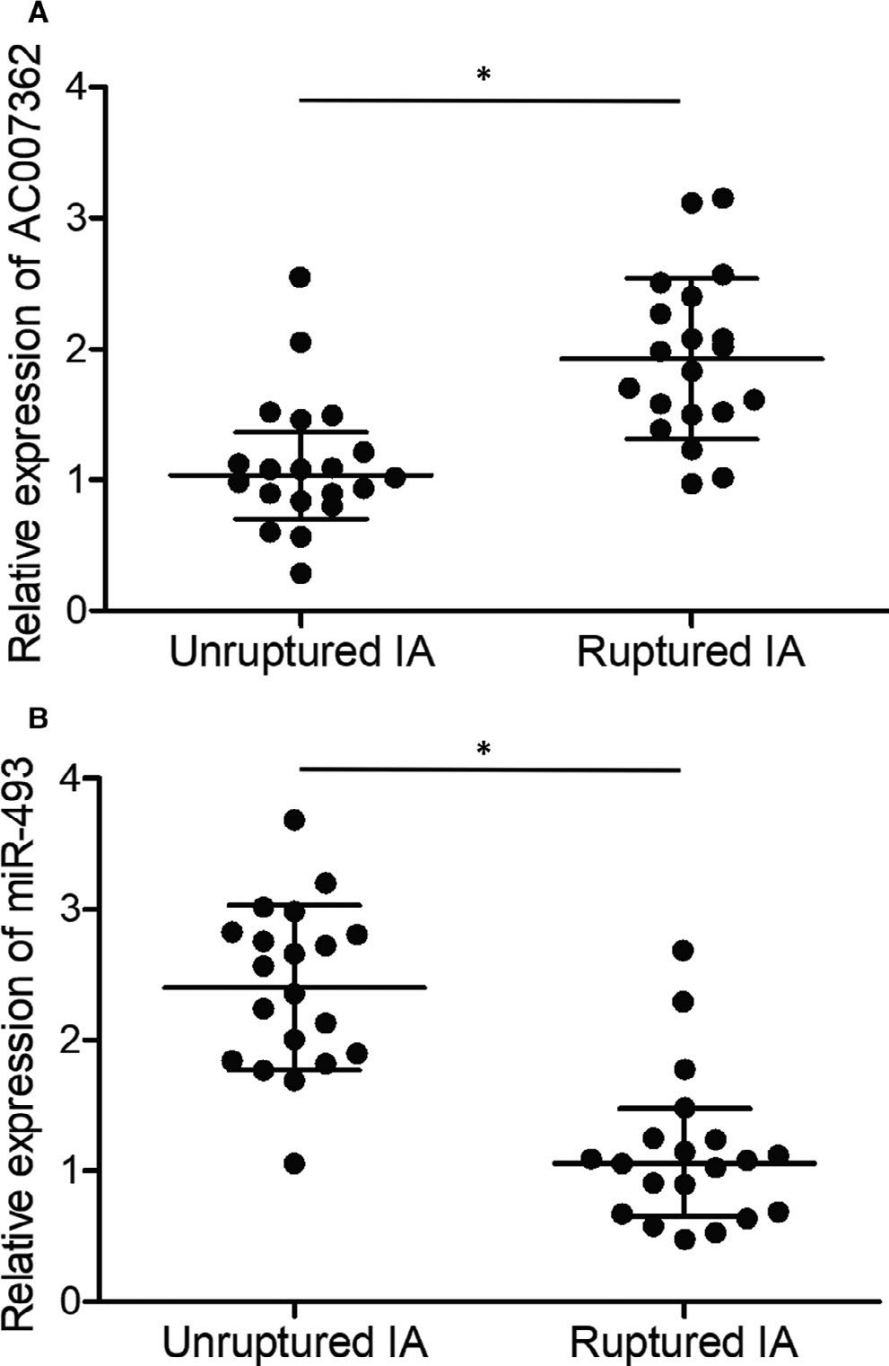
Differential expression of AC007362 and miR‐493 in endothelial cells isolated from unruptured and ruptured IA (*P value <.05 compared with unruptured IA group). (A) Enhanced expression of AC007362 was observed in endothelial cells isolated from ruptured IA. (B) Suppressed expression of miR‐493 was observed in endothelial cells isolated from ruptured IA

Furthermore, the expression of MCP‐1, AC007362 and miR‐493 was examined in the groups of unruptured and ruptured IA. Both plasma MCP‐1 and cellular AC007362 expression was notably elevated in the group of ruptured IA (Figures 7 and 8A). However, the expression of cellular miR‐493 was obviously decreased in the group of ruptured IA (Figure 8B).

## 4 DISCUSSION

SAH is a form of stroke which is associated with high mortality and morbidity and the pathogenesis of SAH is primarily caused by the rupture of IA. As the suppression of proinflammatory factors apparently reduces both the prevalence and the progression of IA, the presence of inflammation in intracranial artery is indispensable to the pathogenesis of IA.^26^ Nevertheless, the hemodynamic features of IA have not been completely clarified. For example, in the dome of an expanding IA, the area with a low WSS apparently overlaps with the region undergoing expansion, indicating that the low WSS value is conductive to the expansion of IA.^34^, ^35^, ^36^ Nevertheless, given the fact that a previous cohort study has demonstrated clear correlation between the volume of an IA and its risk of rupture,^37^ which means the bigger size of the IA, the more risk of rupture of the aneurysm. While during the clinical practice, we do notice there does exist such correlation relationship between the size and the risk of rupture, while this is not always the case, we also do notice some unruptured IA cases with relative bigger diameter, while others ruptured IA cases with relative small size of the aneurysm. In this study, we collected and compared between such extreme cases (bigger unruptured cases vs smaller ruptured cases) to determine the molecular mechanism underlysing.

The endothelial cells covering the wall of blood vessels can respond to shear stress to maintain the homeostasis of blood circulation. The effects of shear stress on the interaction between endothelial cells and blood as well as on the activity of junction molecules and expression of genes involved in the pathogenesis of development as well as the rupture of IA have been well documented.^38^, ^39^, ^40^ In fact, shear stress can up‐regulate the expression of MMP‐1, Tie2 and VEGF in endothelial cells and stimulate the migration of endothelial cells which might be responsible for the development and rupture of IA.^41^, ^42^ In this study, we established a cellular model to evaluate the effect of shear stress on the expression of a group of lncRNAs. We found that AC007362 was significantly up‐regulated in HUVECs exposed to shear stress. Meanwhile, we found that the expression of miR‐493 was decreased while the expression of MCP‐1 was elevated obviously in HUVECs exposed to shear stress. A few shear‐induced events may be responsible for triggering the positive‐feedback circle of CCN1/α6β1‐NFκB. First, shear‐induced deposition of fibronectin occurs soon after the exposure of ECs to turbulent flow.^43^ Then, αvβ3 of ECs binds to fibronectin in the provisional matrix to mediate flow‐induced activation of NF‐κB as well as the generation of proinflammatory responses.^44^

IIli et al^45^ were the first to clarify the molecular mechanism underlying stress‐induced histone modifications in endothelial cells. Other experiments carried out on growth factor‐induced remodelling of chromatin indicated that while both growth factors and shear stress can share the same signalling cascade, the modifications of histone induced by growth factors and shear stress are different from each other.^46^ In this study, we performed RRBS to analyse the level of DNA methylation of AC007362 promoter. The results showed that the level of DNA methylation of AC007362 promoter and the expression of DNMT1 were both decreased by shear stress.

MCP‐1 plays a critical role in neuro‐inflammation.^47^ In the human brain, microglia are the primary carrier of MCP‐1 as well as its receptor MCP‐1.^47^ While inflammatory reactions can prevent disease‐induced tissue damages in certain cases, chronic inflammatory reactions can exert a cytotoxic effect to aggravate the condition of many diseases. An increased level of MCP‐1 was found in the brain of patients of alcohol abuse.^48^ In addition, the levels of IL‐1β, TNF‐α, MCP‐1, and IL‐6 expression in serum were increased in IA patients.^49^, ^50^ MCP‐1 can also promote the infiltration of macrophages as well as the adhesion of macrophages onto the wall of IA artery.^51^ IA rupture can also be induced by Ets‐1, which is a regulator of vascular inflammation.^52^ In this study, we enrolled a group of IA patients and divided them into groups of unruptured and ruptured IA, respectively. The DNMT1 expression in the group of unruptured IA was higher than that in the group of ruptured IA. As a result, the level of DNA methylation of AC007362 promoter was also higher in the group of unruptured IA. Besides, miR‐493 expression was up‐regulated while MCP‐1 expression was inhibited in the group of unruptured IA.

MCP‐1 is a type of chemokine with the ability to attract monocytes as well as to induce the inflammation in gout. In human genome, the promoters of about half of protein‐coding genes are hypo‐methylated under normal conditions. Nevertheless, the expression of human genes is often impaired by the hypermethylation of their promoters.^53^ A previous study demonstrated that the hypo‐methylation of the MCP‐1 gene increases the risk of gout.^54^ In addition, an apparently decreased level of MCP‐1 promoter methylation has been found in patients with gout. Thus, it has been speculated that the hypo‐methylation of MCP‐1 promoter may increase its expression to promote the pathogenesis of gout.^55^

However, despite that human subjects were used in our study, the small sample size was one of the limitations of our study. To further explore the molecular mechanisms proposed in our study, larger sample size is necessary in our subsequent study, which is more appropriate for the determination of IA rupture risk among the patient groups.

## 5 CONCLUSION

The above data showed that low WSS in conjunction with turbulent flow can induce the expression of MCP‐1 in endothelial cells, which in turn promotes sustained macrophage infiltration during the progression of IA and increases the risk of IA rupture.

## CONFLICT OF INTEREST

None.

## AUTHOR CONTRIBUTIONS

**Cheng Chu:** Conceptualization (equal); investigation (equal); supervision (equal); writing – original draft (equal). **Gang Xu:** Conceptualization (equal); investigation (equal); project administration (equal); writing – original draft (equal). **Xiaocong Li:** Investigation (equal); resources (equal); software (equal). **Zuowei Duan:** Investigation (equal); resources (equal); visualization (equal). **Lihong Tao:** Investigation (equal); resources (equal); software (equal). **Hongxia Cai:** Investigation (equal); resources (equal); validation (equal). **Ming Yang:** Validation (equal); visualization (equal). **Xinjiang Zhang:** Validation (equal); visualization (equal). **Bin Chen:** Methodology (equal); visualization (equal). **Yanyu Zheng:** Formal analysis (equal); investigation (equal). **Hongcan Shi:** Funding acquisition (equal); supervision (equal); writing – review & editing (equal). **Xiaoyu Li:** Formal analysis (equal); writing – review & editing (equal).

## ETHICAL APPROVAL

The Human Research Ethics Committees of Affiliated Hospital of Yangzhou University has approved this research (Approval Number: YZDXFS153362) and all methods were performed in accordance with the last vision of the Declaration of Helsinki.

## INFORMED CONSENT

Written informed consent was obtained from all patients before the initiation of this study.

## Data Availability

The data that support the findings of this study are available from the corresponding author upon reasonable request.
